# When performance declines: culturally mediated delay in pediatric knee fracture following traditional healing and scarification—a case report from rural Cameroon

**DOI:** 10.3389/fped.2026.1810974

**Published:** 2026-06-10

**Authors:** Ibrahim Npochinto Moumeni, Fred Dikongue Dikongue

**Affiliations:** 1 Department of Physical Therapy & Physical Medicine, Faculty of Medicine and Pharmaceutical Sciences, University of Dschang, Dschang, West Region, Cameroon; 2 Department of Physical Medicine & Osteopathy, Regional Hospital of Bafoussam, Bafoussam, West Region, Cameroon; 3 Institute for Applied Neurosciences and Functional Rehabilitation (INAREF), Odza-Yaoundé, Cameroon; 4 Franco-African Center for Applied Rehabilitation and Health Sciences (CFARASS), Foumbot, West Region, Cameroon; 5 Department of Geriatrics and Gerontology, Sorbonne Université, Pitié-Salpêtrière Hospital, Paris, France; 6 Licensed Practitioner, Paris, France; 7 Faculty of Health Sciences, University of Parakou, Parakou, Benin; 8 French-Speaking African Society for Neurorehabilitation (SAFNeR), Parakou, Benin; 9 UREKIM—Research Unit in Physiotherapy and Physical Medicine, Faculty of Medicine and Pharmaceutical Sciences, University of Dschang, Dschang, West Region, Cameroon; 10 Centre de Recherche en Santé Humaine et Développement des Médicaments (CRESHDEM), Faculty of Medicine and Pharmaceutical Sciences, University of Dschang, Dschang, West Region, Cameroon; 11 Faculty of Medicine and Biomedical Sciences, University of Douala, Douala, Cameroon

**Keywords:** benevolent parental neglect, Cogni-famille protocol, culturally mediated delay, demineralization, disuse osteopenia, knee, neglected injury, pediatric trauma

## Abstract

**Background:**

Delayed presentation of pediatric musculoskeletal trauma remains a critical yet underexplored challenge in sub-Saharan Africa, where cultural beliefs, socioeconomic constraints, and reliance on traditional healing practices frequently postpone biomedical consultation. The functional and neurobehavioral consequences of such delays in the pediatric population have received limited scientific attention.

**Case presentation:**

We report the case of a 17-year-old male adolescent from Foumbot, a rural community in the West Region of Cameroon, who sustained a left knee injury during a neighborhood football match. Due to financial hardship and culturally rooted health beliefs, the family initially sought traditional care, including repeated massage sessions and therapeutic scarification around the knee. No biomedical consultation was pursued for three months. The patient was eventually referred to the Physical Medicine and Rehabilitation Unit of the Regional Hospital of Bafoussam after his football coach identified a progressive decline in athletic performance and encouraged medical evaluation. Clinical examination revealed limited knee range of motion (active flexion 75°, extension deficit 15°), pain on weight-bearing (Visual Analog Scale 7/10), visible scarification scars, muscular deconditioning, and kinesiophobia. Radiographic evaluation revealed significant regional demineralization of the distal femur and proximal tibia consistent with disuse osteopenia, along with cortical irregularities suggestive of a neglected traumatic injury with delayed consolidation. Baseline functional assessment using the Lysholm Knee Score (41/100, “poor”) and Tegner Activity Scale (level 2) documented significant functional impairment.

**Intervention:**

A seven-week progressive rehabilitation program was implemented, structured in three phases: (1) therapeutic education and cultural alignment with family co-therapy training; (2) progressive mobilization with task-oriented motor training and proprioceptive exercises; (3) sport-specific reintegration with confidence restoration strategies. Parents were actively involved as co-therapists following the previously validated Cogni-Famille model.

**Outcomes:**

At seven weeks, the Lysholm Knee Score improved from 41 to 87/100 (“good”), the Tegner Activity Scale from level 2 to level 7, pain decreased from 7/10 to 1/10, active flexion reached 130°, and extension deficit resolved. The patient returned to competitive football without significant limitation.

**Conclusion:**

This case illustrates how culturally mediated therapeutic delay, driven by the convergence of benevolent parental neglect, traditional healing reliance, and socioeconomic barriers, can lead to significant functional impairment in pediatric trauma. The observation that consultation was triggered by performance decline detected by a community actor rather than by pain or parental initiative underscores the potential role of sports coaches, teachers, and community leaders in early detection and referral. Culturally sensitive rehabilitation strategies, family-centered care, and community-based screening represent promising approaches to improve pediatric trauma outcomes in low-resource settings.

## 1 Introduction

Pediatric musculoskeletal trauma constitutes a significant public health burden, with fractures accounting for a substantial proportion of injuries in children and adolescents during sports and recreational activities (1, 2). The knee, as the most mechanically complex articulation of the lower limb, is particularly vulnerable during adolescence, when physeal vulnerability and increasing athletic demands converge (3). Timely diagnosis and appropriate management are essential to prevent chronic pain, joint stiffness, growth disturbance, and long-term functional limitation (4).

Yet delayed presentation of pediatric fractures remains disproportionately frequent in low- and middle-income countries, where healthcare-seeking behaviors are shaped by socioeconomic, geographical, and cultural determinants (5, 6). In sub-Saharan Africa, families commonly rely on traditional massage, herbal treatments, or scarification as first-line approaches to pediatric musculoskeletal injury, guided by culturally rooted beliefs regarding pain and the perceived self-limiting nature of childhood trauma (7, 8). Although these practices fulfill an important sociocultural role, they may inadvertently delay biomedical consultation and increase the risk of neglected or complicated injuries (9). Scarification in particular—a practice involving deliberate skin incisions for therapeutic purposes, widely employed in rural communities of Central and West Africa—carries documented risks of diagnostic masking and treatment delay (10, 11).

In this context, we previously introduced the concept of Benevolent Parental Neglect (BPN) to describe diagnostic delay resulting from well-intentioned parents who minimize pediatric symptoms due to cultural interpretations of pain, compounded by economic barriers to care (12). Beyond the orthopedic implications, prolonged untreated injury in the pediatric population may trigger neurofunctional maladaptation including altered motor control, kinesiophobia, and sensorimotor disruption during critical developmental periods (13, 14)—consequences that contemporary neuroplasticity research demonstrates can have lasting effects on functional capacity (15, 16). An additional underinvestigated dimension is the role of community actors such as sports coaches and teachers as informal health gatekeepers capable of detecting functional decline and prompting medical referral (17).

We present the case of a 17-year-old adolescent from rural Cameroon with a neglected traumatic knee injury following three months of exclusive traditional healing, including massage and scarification, in whom hospital consultation was prompted not by pain or parental initiative but by a decline in athletic performance identified by his football coach. This case illustrates the concept of culturally mediated therapeutic delay, extends the BPN framework to include the traditional healing pathway, and highlights the role of community-based detection and culturally sensitive rehabilitation in improving pediatric trauma outcomes in low-resource settings.

## 2 Case presentation

### 2.1 Administrative data and ethical considerations

Written informed consent was obtained from both parents for the anonymized publication of clinical data, radiographic images, and outcome measures. This retrospective clinical evaluation was conducted under the Institutional Certification N°43/DRSO/HRB/55/2023 issued by the Regional Hospital of Bafoussam, which authorizes retrospective clinical evaluations based exclusively on anonymized data obtained from routine patient care under the direct coordination and oversight of the responsible department chief, in accordance with prevailing regulations of the Cameroon healthcare system, the Declaration of Helsinki, and international ethical standards. All identifying information was removed to ensure patient confidentiality. This report was prepared in accordance with the CARE (CAse REport) reporting guidelines (18).

### 2.2 Sociocultural and socioeconomic context

The patient came from a low-income household in a rural farming community. Both parents were subsistence farmers with irregular financial resources and limited access to specialized healthcare facilities. Transportation costs (estimated at 5,000–10,000 XAF per round trip), loss of working days, and perceived hospital expenses constituted major barriers to early biomedical consultation.

In this rural setting, traditional medicine remains a prevalent and culturally legitimate first-line option for musculoskeletal injuries. Cultural beliefs commonly frame post-traumatic pain in adolescents as temporary, growth-related, or amenable to traditional healing, leading to delayed or absent medical care. This sociocultural framework is consistent with the previously described concept of Benevolent Parental Neglect (BPN), in which parents unintentionally minimize symptoms due to cultural interpretations, economic constraints, and trust in traditional healing systems (12).

### 2.3 History of present illness

The injury occurred during a neighborhood football match when the patient fell onto his left lower limb following a collision with another player. He reported immediate pain and inability to bear full weight, but no medical consultation was sought. Instead, the family consulted a local traditional healer (tradipraticien) within the first week following injury.

The patient underwent a course of repeated traditional massage sessions over the following weeks. Additionally, multiple therapeutic scarifications were performed around the knee region, based on the cultural belief that these incisions could “release trapped pain,” “drain bad blood,” and facilitate bone healing. The traditional healer also applied herbal poultices following the scarification procedures.

Despite persistent pain, progressive joint stiffness, and increasing functional limitation, symptoms were minimized by both the family and the traditional healer. The adolescent progressively reduced participation in sports and recreational activities, adopting compensatory strategies for daily activities including a marked antalgic gait and avoidance of stairs and uneven terrain.

Three months after the initial trauma, the patient's football coach observed a marked and progressive decline in athletic performance: the patient avoided running, showed reluctance during contact situations, demonstrated asymmetric movement patterns, and exhibited visible lack of confidence during training sessions. Concerned about a potential unresolved injury, the coach personally recommended hospital evaluation and accompanied the patient and his parents to the Regional Hospital of Bafoussam. Parental consent for biomedical care was obtained only after this community-driven referral, illustrating the critical role of community actors in facilitating access to rehabilitation services in low-resource settings.

### 2.4 Parental and community testimonies

Mother (farmer, 42 years): “At first, we thought it was just a minor injury. In our culture, boys are expected to be strong and continue. We believed the pain would resolve with the healer's treatments.”

Father (farmer, 47 years): “We did not have money for hospital care. The healer told us that massage and scarification would heal the bone. We trusted his experience.”

Football coach: “I noticed he was no longer the same player. He avoided contact, limped during sprints, and seemed afraid. His performance dropped sharply over several weeks. I told the parents this was not normal and insisted he be taken to the hospital.”

These testimonies illustrate the complex interaction between cultural health beliefs, economic constraints, trust in traditional healing, and the catalytic role of community awareness in determining healthcare-seeking trajectories.

### 2.5 Clinical examination at presentation

Physical examination at three months post-injury revealed the following findings:

**Inspection:** Mild periarticular swelling of the left knee. Multiple linear and punctiform scarification scars distributed over the anteromedial and lateral aspects of the knee, consistent with traditional therapeutic scarification. No signs of active infection. Quadriceps amyotrophy of the left thigh with a measured circumference difference of 2.5 cm compared to the contralateral side (measured 15 cm above the superior patellar pole).

**Range of motion:** Active flexion limited to 75° (contralateral: 140°). Extension deficit of 15° (inability to achieve full extension). Passive flexion reached 85° with a firm, painful end-feel.

**Pain assessment:** Visual Analog Scale (VAS) at rest: 3/10. VAS on weight-bearing and during active mobilization: 7/10. Pain localized primarily to the distal femoral and periarticular region.

**Functional assessment:** Antalgic gait pattern with shortened stance phase on the affected side. Inability to perform single-leg stance. Unable to negotiate stairs without handrail support. Unable to run, jump, or perform any sport-specific activity.

**Neurovascular status:** No distal vascular or neurological deficit identified. Peripheral pulses intact. Sensation preserved in all dermatomes.

**Psychobehavioral assessment:** Marked kinesiophobia with apprehension during passive mobilization. Fear of re-injury. Social withdrawal from sports activities. Decreased self-esteem related to performance decline.

### 2.6 Baseline functional scoring

Standardized functional assessment was performed at initial evaluation using validated instruments for knee pathology in the active population Tables 1, 2:

**Table 1:** Lysholm knee score: detailed component analysis at baseline (week 0) and post-rehabilitation (week 7).

Lysholm knee score component	Baseline (Week 0)	Post-rehabilitation (Week 7)
Limp	3 (moderate limp)	5 (no limp)
Support	5 (no support needed)	5 (no support needed)
Stair climbing	2 (one step at a time)	10 (no problems)
Squatting	0 (impossible)	4 (slightly impaired)
Instability	10 (never gives way)	25 (never gives way)
Pain	10 (marked, during activity)	20 (slight, during activity)
Swelling	6 (on ordinary exertion)	10 (none)
Locking	5 (catching, no locking)	15 (no locking)
Total score	41/100 (“Poor”)	87/100 (“Good”)

Each component reflects a specific functional domain. The total score improved from 41/100 (“poor”) to 87/100 (“good”), representing a 46-point gain exceeding the established minimal clinically important difference (MCID) of 10–12 points (27, 28).

**Table 2:** Tegner activity scale: baseline, post-rehabilitation, and pre-injury activity levels.

Tegner activity scale	Score
Baseline (Week 0)	Level 2 (walking on uneven ground possible; light housework)
Post-rehabilitation (Week 7)	Level 7 (competitive sports: football, athletics at recreational level)
Pre-injury level (reported)	Level 8 (competitive football, regular training)

The scale ranges from 0 (disability) to 10 (elite competitive sport). The five-level improvement (level 2 to level 7) documents the transition from severe activity restriction to competitive recreational sports participation, approaching the patient's pre-injury level of 8 (19, 20).

The Lysholm Knee Score of 41/100 at baseline classified the patient's knee function as “poor,” reflecting significant impairment across multiple domains. The Tegner Activity Scale score of 2 (compared to a pre-injury level of 8) documented a dramatic reduction in activity capacity. These validated scores provide objective evidence of the functional burden of the three-month diagnostic delay and serve as quantitative endpoints for rehabilitation monitoring (19, 20).

### 2.7 Radiological findings

**Figure 1:**
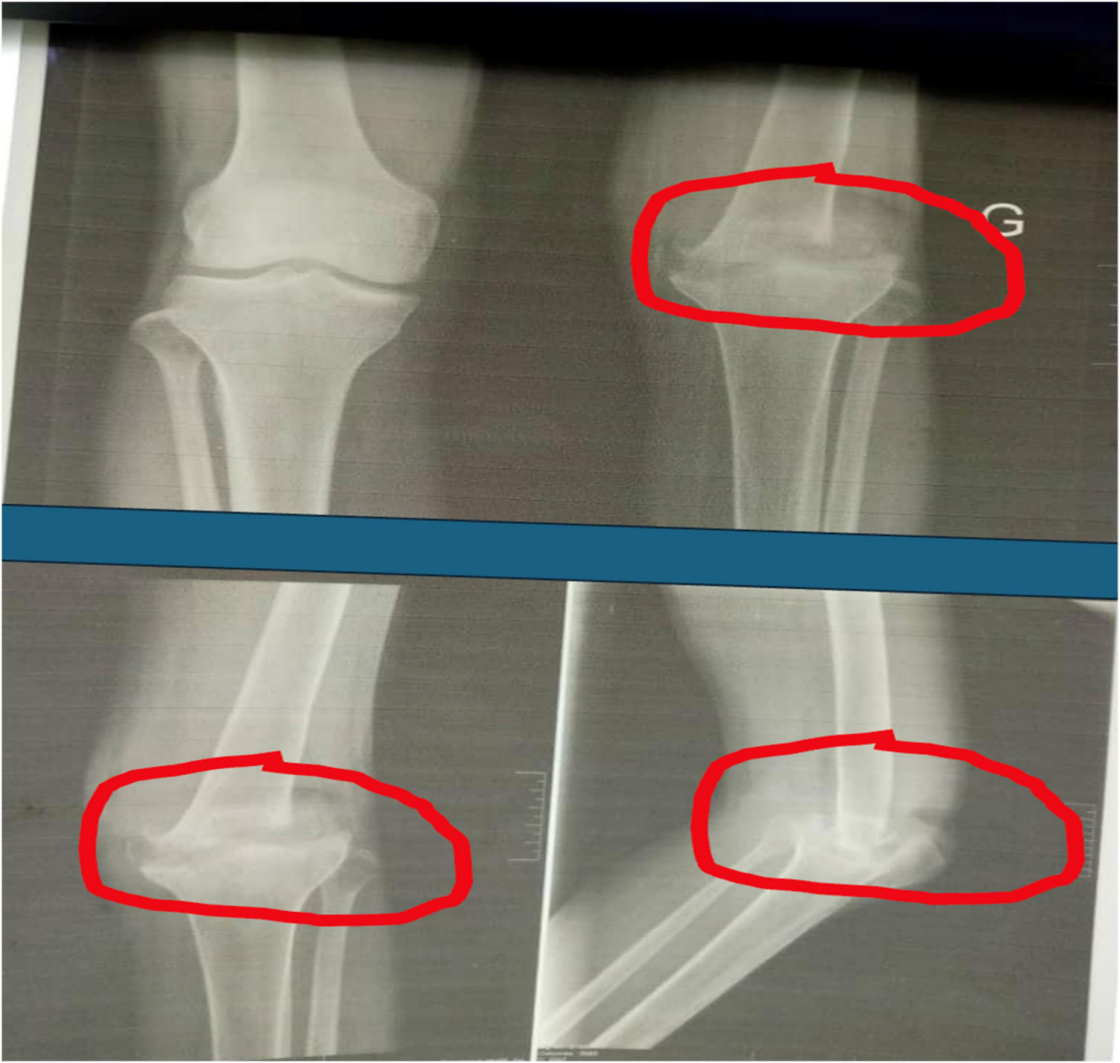
Anteroposterior and lateral radiographs of the left knee in a 17-year-old male, three months after untreated sports-related trauma. Red circles highlight areas of significant regional demineralization (disuse osteopenia) involving the distal femur and proximal tibia, with cortical irregularities at the distal femoral metaphysis consistent with a healing traumatic lesion. Note the diffuse reduction in bone density in the periarticular regions compared to adjacent diaphyseal segments, reflecting three months of functional underloading. “G” marker indicates left side (Gauche). No significant articular displacement or physeal arrest is observed.

Standard anteroposterior and lateral radiographs of the left knee (Figure 1) were obtained and demonstrated:

Significant regional demineralization (osteopenia) involving the distal femoral metaphysis and epiphysis as well as the proximal tibial metaphysis, manifested by diffuse reduction in bone density compared to the adjacent diaphyseal segments. This pattern of periarticular osteopenia is consistent with disuse-related bone demineralization secondary to three months of functional underloading and weight-bearing avoidance. Cortical irregularities were noted at the distal femoral metaphysis, suggestive of a healing traumatic lesion with delayed consolidation. No significant articular surface disruption, displacement, or physeal arrest was identified. The growth plates (physes) appeared open, consistent with the patient's age. No foreign bodies, periosteal reaction suggestive of infection, or signs of osteomyelitis were observed. The overall mechanical alignment of the lower limb was preserved.

#### 2.7.1 Radiological differential diagnosis

Given the clinical context and radiographic pattern, the following differential diagnoses were systematically considered and addressed: (1) Metaphyseal fracture (most probable retained diagnosis): the cortical irregularities at the distal femoral metaphysis, combined with the history of acute traumatic loading, periarticular disuse osteopenia, and three-month functional avoidance, are most consistent with a partially consolidated metaphyseal fracture. The absence of cortical displacement, periosteal reaction beyond consolidation lines, and physeal bridging supports a non-displaced or minimally displaced fracture with delayed union secondary to inadequate immobilization. (2) Physeal injury (Salter-Harris type): excluded on the basis of open, morphologically normal growth plates bilaterally on both AP and lateral projections, without physeal widening, step-off, or epiphyseal displacement. (3) Avulsion fracture: the absence of discrete cortical fragment, bony prominence defect, or ligamentous tension point involvement on standard views argues against an isolated avulsion injury, though micro-avulsion cannot be entirely excluded without MRI. (4) Occult fracture: given the resource-limited context, MRI and CT were not available. However, the combination of acute traumatic onset, persistent weight-bearing limitation, periarticular demineralization consistent with disuse, and cortical irregularity on plain films provides sufficient clinical and radiological justification for the working diagnosis of neglected traumatic injury with delayed consolidation. (5) Osteomyelitis and septic arthritis: specifically excluded by absence of periosteal reaction beyond consolidation patterns, normal soft tissue planes, afebrile course, and no systemic inflammatory signs throughout the three-month delay period. (6) Bone tumor: excluded by the benign-appearing pattern of demineralization (periarticular, uniform, consistent with disuse), absence of aggressive periosteal reaction, cortical destruction, or soft tissue mass.

The retained diagnosis of non-displaced or minimally displaced distal femoral metaphyseal fracture with delayed consolidation is supported by the mechanistic coherence of the clinical history (acute traumatic axial loading in a skeletally immature patient), the radiographic findings (periarticular disuse osteopenia, metaphyseal cortical irregularity, preserved physis), and the functional trajectory (progressive deconditioning consistent with three months of pain-mediated weight-bearing avoidance). This interpretation is further corroborated by the favorable response to progressive, controlled loading during rehabilitation, with restoration of full range of motion and return to competitive sport, consistent with fracture consolidation under guided physiological stress. A post-treatment radiograph was planned at the 12-week follow-up visit to document osseous consolidation; however, the family was unable to attend the scheduled appointment due to transportation cost constraints—a recurrent barrier documented in this socioeconomic context (5, 6). Three-month telephone follow-up confirmed complete functional recovery with no pain recurrence, asymmetry, or re-injury, providing indirect clinical evidence of satisfactory consolidation.

The radiographic pattern of regional demineralization provides objective evidence of the functional consequences of the three-month culturally mediated therapeutic delay: prolonged partial weight-bearing avoidance and disuse led to measurable bone density loss, paralleling the clinically observed muscular deconditioning and joint stiffness Figure 1. This finding underscores that delayed trauma management generates not only soft tissue consequences but also quantifiable skeletal changes that may increase the risk of secondary fracture during rehabilitation and return to sport (15).

### 2.8 Diagnosis

Based on the clinical history, physical examination, functional assessment, and radiological findings, the diagnosis was established as: neglected traumatic knee injury of the left lower limb with significant regional demineralization (disuse osteopenia) of the distal femur and proximal tibia, cortical irregularities suggestive of a healing traumatic lesion, secondary joint stiffness, quadriceps deconditioning, antalgic gait pattern, kinesiophobia, and functional disability, in the context of a three-month culturally mediated therapeutic delay following exclusive traditional healing management including scarification.

## 3 Intervention

A structured, progressive rehabilitation program was designed and implemented over seven weeks, incorporating evidence-based principles of progressive mobilization, task-oriented motor training, neuroplasticity-informed exercise prescription, and culturally sensitive family-centered care. The program was structured in three sequential phases with specific therapeutic objectives and dosimetry.

### 3.1 Phase 1: therapeutic education and cultural alignment (weeks 1–2)

The initial phase prioritized patient and family education, therapeutic alliance building, and respectful cultural mediation. Key elements included:

**Patient and family education:** A detailed, culturally adapted explanation of the injury mechanism, fracture biology, and the rationale for progressive rehabilitation was provided using visual aids and local-language communication. The concept of “rehabilitation as medicine” (21) was introduced to frame the rehabilitation process within a pharmacological paradigm familiar to the family.

**Cultural mediation:** Traditional healing practices were addressed respectfully, without judgment or confrontation. The therapeutic team acknowledged the cultural legitimacy of the family's initial choices while explaining the complementary role of structured rehabilitation. This approach aligns with the previously described concept of “therapeutic pluralism” (22), which recognizes the coexistence of biomedical and traditional healing systems.

**Family co-therapy training:** Both parents were trained as co-therapists, following the previously validated Cogni-Famille model adapted from post-stroke neurorehabilitation (23). Parents received hands-on instruction in supervised range-of-motion exercises, pain monitoring techniques, and daily exercise documentation. This family-mediated approach enabled a therapeutic dose exceeding hospital-based sessions alone, targeting a minimum of 45 min of daily home-based structured exercise.

**Initial pain management:** Gentle, pain-free active-assisted mobilization was initiated. Cryotherapy was applied post-session. Analgesic medication was prescribed as needed in coordination with the referring physician.

### 3.2 Phase 2: progressive mobilization and functional rehabilitation (weeks 3–5)

The second phase focused on progressive restoration of joint range of motion, muscular reconditioning, proprioceptive training, and task-oriented functional recovery.

**Progressive mobilization:** Active and active-assisted knee flexion-extension exercises were performed with incremental range progression (target: 10° flexion gain per week). End-range stretching was introduced with sustained holds (30 s, 5 repetitions per session) following contemporary evidence on optimal stretching parameters for post-traumatic stiffness (24).

**Muscular reconditioning:** Open and closed kinetic chain strengthening exercises targeting the quadriceps, hamstrings, and hip stabilizers were implemented with progressive resistance. Initial exercises included isometric quadriceps sets, straight-leg raises, and mini-squats, progressing to step-ups, leg press simulation, and functional lunges.

**Proprioceptive training:** Single-leg balance exercises on stable then unstable surfaces were introduced, following the principle of proprioceptive-directed plasticity (25). These exercises targeted the restoration of sensorimotor integration and movement confidence, addressing the observed kinesiophobia.

**Task-oriented training:** Functional activities replicating daily tasks (stair climbing, walking on uneven terrain, sit-to-stand transfers) were incorporated to promote meaningful motor recovery and cortical activation through ecologically valid movement patterns (26).

**Therapeutic dose:** Three supervised hospital sessions per week (60 min each) supplemented by daily home-based exercises (45–60 min) supervised by trained parents, achieving a total therapeutic exposure of approximately 7–8 h per week.

### 3.3 Phase 3: sport-specific reintegration and confidence restoration (weeks 6–7)

The final phase targeted sport-specific functional recovery, psychological readiness, and safe return to athletic participation.

**Sport-specific exercises:** Progressive running program (walk-jog-run intervals), agility drills, ball-handling exercises, and sport-specific movement patterns adapted from the patient's football activities were introduced. Training intensity was guided by pain response (VAS < 3/10 threshold) and movement quality assessment.

**Confidence restoration:** Graded exposure to sport-specific scenarios addressed kinesiophobia. The coach was integrated into the rehabilitation process, providing supervised, graduated return-to-play sessions. This coach-rehabilitation partnership reinforced the community detection model by closing the feedback loop between initial referral and functional reintegration.

**Discharge criteria:** The patient was considered ready for progressive return to competitive sport when the following criteria were met: pain VAS ≤ 2/10 during sport-specific activity, knee flexion ≥ 125° with full extension, Lysholm score ≥ 80/100, Tegner level ≥ 6, and subjective confidence in sport-specific movement patterns.

## 4 Outcomes

Functional outcomes were assessed at baseline (Week 0) and at completion of the rehabilitation program (Week 7) using standardized clinical measurements and validated functional instruments Table 3.

**Table 3:** Comprehensive outcome summary: clinical, functional, and psychobehavioral parameters at baseline (week 0) versus post-rehabilitation (week 7).

Parameter	Baseline (W0)	Post-rehab (W7)	Change
Active knee flexion	75°	130°	+55°
Extension deficit	15°	0°	Resolved
VAS pain (activity)	7/10	1/10	−6 points
VAS pain (rest)	3/10	0/10	−3 points
Lysholm Knee Score	41 (Poor)	87 (Good)	+46 points
Tegner Activity Scale	Level 2	Level 7	+5 levels
Thigh circumference diff.	2.5 cm	0.8 cm	−1.7 cm
Single-leg stance	Impossible	30 s stable	Restored
Stair climbing	With handrail only	No difficulty	Normalized
Running capacity	Impossible	Full sprint	Restored
Sport participation	None	Competitive football	Returned

Data include range of motion, pain (Visual Analog Scale), validated functional scores (Lysholm, Tegner), muscular reconditioning markers, and sport-specific capacity. All parameters demonstrated clinically meaningful improvement following a seven-week progressive rehabilitation program integrating family co-therapy.

The Lysholm Knee Score improved from 41/100 (“poor”) to 87/100 (“good”), representing a 46-point improvement that substantially exceeds the minimal clinically important difference (MCID) of 10–12 points established in the literature for knee pathology (27, 28). This improvement reflects meaningful functional recovery across all Lysholm domains, with the most notable gains in pain, squatting ability, stair climbing, and subjective stability.

The Tegner Activity Scale improved from level 2 (limited walking activities) to level 7 (competitive recreational sports), approaching the patient's pre-injury level of 8. This five-level improvement documents the successful transition from severe activity restriction to functional sports reintegration over the seven-week rehabilitation period (20).

Active knee flexion improved by 55° (from 75° to 130°), and the 15° extension deficit resolved completely, restoring near-normal range of motion. Pain decreased from 7/10 to 1/10 during activity, enabling progressive weight-bearing and sport-specific training. Quadriceps reconditioning reduced the thigh circumference difference from 2.5 cm to 0.8 cm.

From a psychobehavioral perspective, the patient demonstrated resolution of kinesiophobia, restoration of movement confidence, and renewed social engagement in team sports. At the final assessment, the patient had returned to regular football training and expressed readiness for competitive participation.

A three-month telephone follow-up confirmed sustained functional gains, continued sports participation without pain recurrence, and improved family awareness regarding the importance of timely biomedical consultation for future injuries.

## 5 Discussion

### 5.1 Culturally mediated therapeutic delay: a multidimensional framework

This case provides clinical evidence for the concept of culturally mediated therapeutic delay (CMTD) in pediatric musculoskeletal trauma. Unlike simple delayed presentation, CMTD describes a multifactorial process in which structural, economic, cultural, and interpersonal determinants converge to postpone biomedical consultation in favor of traditional healing pathways. In this framework, delay is not an isolated event but a trajectory shaped by culturally coherent health behaviors (6, 7, 29).

It is important to clarify the epistemological status of the conceptual frameworks developed in this report. CMTD, Benevolent Parental Neglect (BPN), the community detection model, and the late functional plasticity window hypothesis are presented as exploratory theoretical constructs grounded in clinical observation rather than as validated, fully established frameworks. Their purpose is to provide an organized interpretive lens for the reported case and to generate testable hypotheses for future research, consistent with the recognized heuristic function of case-report-derived conceptual proposals in clinical literature (18). These constructs should not be conflated with empirically validated models requiring multicenter replication. Future prospective cohort studies and systematic reviews are necessary to test their generalizability, boundary conditions, and predictive validity across diverse low-resource settings.

The present observation extends the understanding of CMTD by documenting the complete delay trajectory: from initial injury, through cultural interpretation and traditional healing engagement, to eventual biomedical consultation triggered by community detection. This sequential model has implications for public health interventions aimed at reducing diagnostic delay in pediatric trauma across sub-Saharan Africa.

### 5.2 Extension of the benevolent parental neglect framework

This case builds upon and extends the concept of Benevolent Parental Neglect (BPN) previously introduced in the context of Osgood-Schlatter disease in Cameroonian adolescents (12). In the original BPN framework, diagnostic delay resulted primarily from parental minimization of symptoms influenced by cultural interpretations of pain and growth. The present case demonstrates that BPN operates within a broader ecosystem that includes active engagement with traditional healing systems, economic rationing of healthcare expenditure, and community influence on care-seeking decisions.

Critically, this case illustrates the dynamic and non-linear nature of healthcare-seeking trajectories: parental beliefs were not immutable, but evolved in response to external community pressure. The transition from traditional care to biomedical consultation was catalyzed not by symptom progression or parental initiative, but by the intervention of a community actor. This finding suggests that BPN may be responsive to targeted community-level interventions, offering an actionable entry point for public health strategies.

### 5.3 Traditional healing and scarification: clinical and anthropological implications

Traditional medicine remains the dominant first-line approach to musculoskeletal injury across rural sub-Saharan Africa, with usage rates exceeding 80% in some communities (7, 30). The present case documents therapeutic scarification around the knee as a perceived post-traumatic treatment. While scarification fulfills an important sociocultural role, it carries documented risks of diagnostic masking and treatment delay through the creation of a perceived treatment-in-progress that discourages biomedical consultation (10, 11). This observation supports a model of therapeutic pluralism (22) in which traditional healers are engaged as partners in referral pathways rather than opposed—a strategy that respects cultural frameworks while reducing diagnostic delay (9, 31).

### 5.4 Neurofunctional consequences of delayed pediatric trauma

Beyond orthopedic implications, three months of untreated injury produced a constellation of neurofunctional maladaptation: altered motor control, compensatory gait, quadriceps deconditioning, kinesiophobia, and reduced sensorimotor confidence (13, 14). The learned nonuse paradigm (32) provides a useful conceptual parallel: pain-mediated movement avoidance generated a self-reinforcing cycle of disuse and increasing disability, analogous to patterns described in chronic neurological conditions (33). The favorable response to structured rehabilitation supports the reversibility of these maladaptive changes when neuroplasticity-informed intervention is provided in delayed contexts (15, 16), consistent with the working hypothesis of late functional plasticity windows (21, 25)—a concept warranting prospective investigation in skeletally immature populations.

### 5.5 The sports coach as community health gatekeeper

The most original observation in this case is that biomedical consultation was triggered by athletic performance decline detected by the football coach, not by pain or parental initiative. Sports coaches in low-resource communities maintain regular longitudinal observation of functional capacity and movement quality, positioning them as informal sentinels for unrecognized musculoskeletal pathology (17). This community detection model aligns with WHO recommendations for decentralized rehabilitation and task-shifting strategies (34, 35). Structured educational programs for coaches, teachers, and community leaders focused on basic injury recognition and referral criteria could create a sustainable, cost-effective early detection network.

### 5.6 Culturally sensitive rehabilitation: an integrative approach

The rehabilitation strategy employed in this case was deliberately designed to integrate biomedical evidence with cultural sensitivity. Rather than opposing traditional beliefs, the therapeutic team framed rehabilitation within a culturally coherent narrative, using the concept of “rehabilitation as medicine” to bridge the conceptual gap between traditional healing and structured rehabilitation (21, 36).

**Figure 2:**
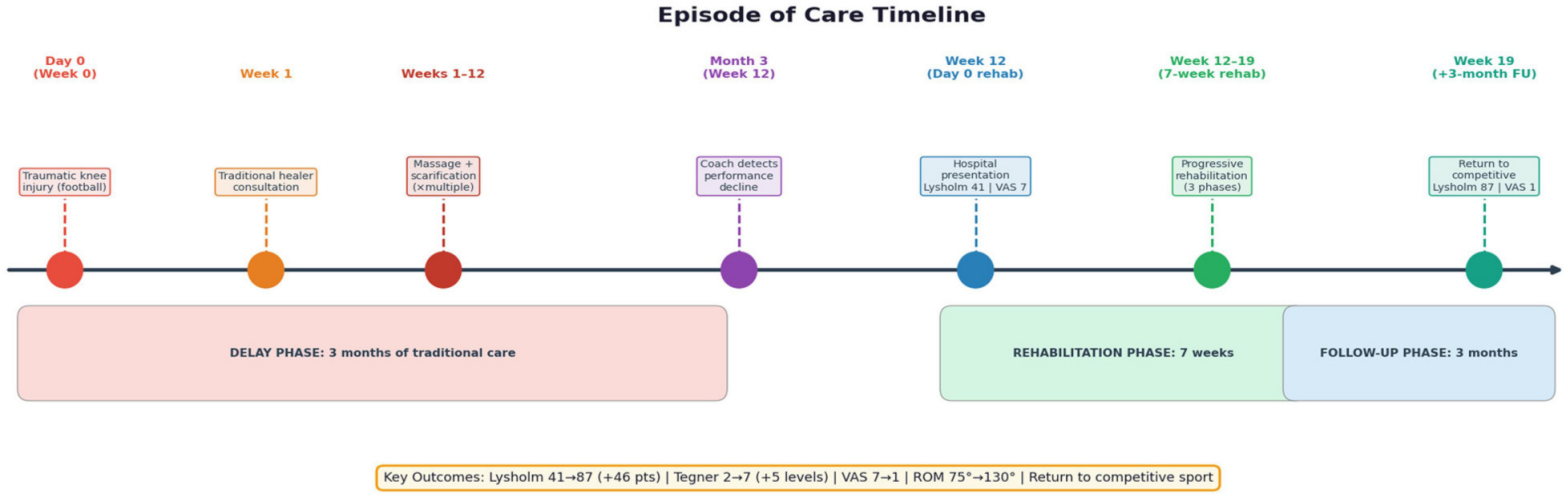
Episode of care timeline illustrating the sequential phases from the index injury to functional recovery. The delay phase (months 0–3) encompasses the period of exclusive traditional care including massage and therapeutic scarification, terminated by community-driven detection of performance decline by the football coach. The rehabilitation phase (weeks 12–19) corresponds to the seven-week structured progressive program implemented at the Regional Hospital of Bafoussam. Key functional outcomes are displayed at each phase transition. FU, follow-up; VAS, Visual Analog Scale; ROM, range of motion.

The integration of parents as co-therapists, adapted from the Cogni-Famille model (23, 37–40), proved particularly effective in this context, Table 4 and Figure 2. By empowering family members as active participants in the rehabilitation process, this approach addressed multiple barriers simultaneously: it reduced dependence on hospital-based sessions, increased total therapeutic dose, reinforced family understanding of the injury, and transformed the family's relationship with biomedical care from passive compliance to active engagement. The improvement from Lysholm 41 to 87 and Tegner 2 to 7 over seven weeks supports the effectiveness of this integrated approach.

**Table 4:** Conceptual comparison between benevolent parental neglect (BPN) and culturally mediated therapeutic delay (CMTD).

Dimension	Benevolent Parental Neglect (BPN)	Culturally Mediated Therapeutic Delay (CMTD)
Definition	Unintentional minimization of pediatric symptoms by loving parents	Systemic delay driven by convergence of cultural, economic, and community factors
Primary agents	Parents/caregivers	Parents, traditional healers, community network
Mechanism	Symptom normalization within family	Active traditional healing pathway preceding biomedical consultation
Trigger for consultation	Symptom worsening	Community detection (coach, teacher, peer)
Traditional healing role	Passive (no treatment)	Active (massage, scarification, herbal treatments)
Functional impact	Diagnostic delay	Neurofunctional maladaptation and participation restriction
Intervention target	Parental education	Community-based detection and culturally sensitive rehabilitation
Public health model	Family-centered awareness	Integrated community health gatekeeper network

BPN, previously introduced by I. Npochinto Moumeni and Atemkeng Tsatedem [12: doi:10.1016/j.hmedic.2026.100419], describes unintentional parental minimization of symptoms. CMTD extends this framework to encompass the broader ecosystem of traditional healing, community dynamics, and socioeconomic determinants that converge to delay biomedical consultation. This conceptual evolution provides an actionable framework for public health interventions targeting pediatric trauma care in sub-Saharan Africa.

### 5.7 Conceptual framework: from BPN to CMTD

Based on this case and our previous work, we propose a conceptual evolution from the original BPN framework to a broader model of Culturally Mediated Therapeutic Delay (Table 4).

### 5.8 Counterfactual analysis: what if medical care had been sought immediately?

A critical question raised by this observation is: what would the clinical trajectory have been if biomedical consultation had been obtained immediately following the injury? While this remains inherently speculative, the available evidence allows for a reasoned counterfactual analysis. In the context of a non-displaced or minimally displaced distal femoral metaphyseal fracture in a skeletally immature adolescent, early biomedical management would typically have consisted of appropriate immobilization (plaster cast or functional brace for 4–6 weeks), analgesic prescription, and weight-bearing guidance, followed by supervised rehabilitation targeting range of motion restoration, muscular reconditioning, and safe return to sport—a pathway well established in the pediatric orthopedic literature (4, 41). Under this scenario, consolidation would be expected within 6–8 weeks, with full functional recovery achievable within 3–4 months, including return to competitive sport. The three-month diagnostic delay in this case resulted in (1): progressive disuse osteopenia with measurable bone density loss, increasing the risk of re-fracture during rehabilitation (2); quadriceps deconditioning requiring specific reconditioning protocols (3); kinesiophobia requiring dedicated behavioral and graded exposure interventions (4); compensatory biomechanical adaptations with antalgic gait patterns that required systematic correction; and (5) a total functional impairment burden documented by a Lysholm score of 41/100 at presentation, compared to expected near-normal function had timely care been provided. Critically, the observation that meaningful recovery was achieved within seven weeks of structured rehabilitation—despite a three-month delay—underscores both the reversibility of disuse-related changes in adolescent populations and the efficacy of intensive, family-centered rehabilitation even in significantly delayed presentations. This finding supports the concept of “late functional plasticity windows” (15, 42–45) as a working hypothesis, suggesting that in skeletally immature populations, the capacity for functional recovery may persist well beyond the classical fracture management timeline, provided appropriate rehabilitation is instituted. The conceptual and public health implications of BPN and CMTD in reducing such delays are addressed in the intervention recommendations of Section 5.2.

### 5.9 Limitations

This report presents a single case and findings cannot be generalized. Objective biomechanical assessment (gait analysis, isokinetic testing) was not available due to resource constraints. The Lysholm and Tegner scores, while validated and widely used, have been primarily validated in adult and post-surgical populations, and their psychometric properties in the specific context of neglected pediatric fractures in African settings warrant further investigation. Radiographic assessment was limited to standard views without computed tomography or magnetic resonance imaging. Long-term outcomes beyond three months were assessed by telephone only. A post-treatment radiograph had been planned to document osseous consolidation at the 12-week follow-up; however, the family was unable to attend the scheduled imaging appointment due to transportation cost constraints—a structural barrier documented throughout this case. Indirect evidence of satisfactory consolidation was provided by full functional recovery and return to competitive sport without pain recurrence at three-month telephone follow-up. Future case series in similar contexts should systematically plan post-treatment imaging and consider teleconsultation or community-level radiology strategies to improve radiological documentation despite transportation barriers (5, 6, 44, 46). Future multicenter prospective studies should evaluate the prevalence and determinants of culturally mediated therapeutic delay across diverse settings and assess the effectiveness of community-based detection programs (47–49, 50–53).

## 6 What this study adds

1. Introduces the concept of Culturally Mediated Therapeutic Delay (CMTD) as a multidimensional framework for understanding delayed pediatric trauma care in low-resource settings.
2. Extends the Benevolent Parental Neglect framework to include traditional healing pathways and community dynamics.
3. Provides the first documented case linking therapeutic scarification to diagnostic delay and disuse osteopenia in pediatric knee trauma.
4. Demonstrates that performance decline detected by a community actor, rather than pain or parental concern, can serve as the primary trigger for biomedical consultation.
5. Identifies sports coaches as potential community health gatekeepers for pediatric musculoskeletal injury detection.
6. Provides quantitative evidence (Lysholm +46 points; Tegner +5 levels) supporting the effectiveness of culturally sensitive, family-centered neurofunctional rehabilitation even after significant diagnostic delay.
7. Documents the patient's perspective through verbal testimonies: the patient's evolving experience of kinesiophobia, loss of athletic identity, and progressive restoration of movement confidence and social engagement in team sports provide a first-person account of the psychobehavioral burden of culturally mediated diagnostic delay and the transformative impact of family-centered rehabilitation on subjective recovery.
8. Provides a visual episode of care timeline (Figure 2) clarifying the complete sequential trajectory from injury to return to competitive sport, in compliance with CARE guidelines for case report reporting.

## 7 Conclusion

This case demonstrates that delayed pediatric trauma management in low-resource settings represents not merely a biomedical failure but a complex sociocultural, neurofunctional, and systemic challenge. The convergence of benevolent parental neglect, traditional healing reliance, economic barriers, and limited community health literacy creates a trajectory of culturally mediated therapeutic delay that can result in significant functional impairment in young patients with treatable musculoskeletal injuries.

The original observation that medical consultation was catalyzed by community detection of performance decline—rather than by pain, symptom progression, or parental initiative—underscores the transformative potential of community-based early detection strategies. Sports coaches, teachers, and community leaders represent an untapped resource for pediatric injury surveillance in settings where formal healthcare infrastructure remains insufficient.

Structured, culturally sensitive rehabilitation integrating family co-therapy, progressive neurofunctional training, and respectful cultural mediation can achieve meaningful functional recovery even in delayed presentations, supporting the principle that it is never too late to rehabilitate. Future research should focus on developing and evaluating community-based detection programs, collaborative traditional healer-biomedical referral pathways, and culturally adapted rehabilitation protocols to improve pediatric trauma outcomes across sub-Saharan Africa.

## Data Availability

The raw data supporting the conclusions of this article will be made available by the authors, without undue reservation.
